# Eradication of high viable loads of *Listeria monocytogenes* contaminating food-contact surfaces

**DOI:** 10.3389/fmicb.2015.00733

**Published:** 2015-07-16

**Authors:** Silvia de Candia, Maria Morea, Federico Baruzzi

**Affiliations:** Institute of Sciences of Food Production, National Research Council of ItalyBari, Italy

**Keywords:** ozone processing, foodborne pathogens, cross-contamination, food-contact surfaces, food plant sanitation, psychrotrophic bacteria

## Abstract

This study demonstrates the efficacy of cold gaseous ozone treatments at low concentrations in the eradication of high *Listeria monocytogenes* viable cell loads from glass, polypropylene, stainless steel, and expanded polystyrene food-contact surfaces. Using a step by step approach, involving the selection of the most resistant strain-surface combinations, 11 *Listeria* sp. strains resulted inactivated by a continuous ozone flow at 1.07 mg m^-3^ after 24 or 48 h of cold incubation, depending on both strain and surface evaluated. Increasing the inoculum level to 9 log CFU coupon^-1^, the best inactivation rate was obtained after 48 h of treatment at 3.21 mg m^-3^ ozone concentration when cells were deposited onto stainless steel and expanded polystyrene coupons, resulted the most resistant food-contact surfaces in the previous assays. The addition of naturally contaminated meat extract to a high load of *L. monocytogenes* LMG 23775 cells, the most resistant strain out of the 11 assayed *Listeria* sp. strains, led to its complete inactivation after 4 days of treatment. To the best of our knowledge, this is the first report describing the survival of *L. monocytogenes* and the effect of ozone treatment under cold storage conditions on expanded polystyrene, a commonly used material in food packaging. The results of this study could be useful for reducing pathogen cross-contamination phenomena during cold food storage.

## Introduction

Foodborne outbreaks affect thousands of consumers across the world every year. In 2012, the US Centers for Disease Control and Prevention (CDC, 2014) reported more than 19,500 infections, 4,600 hospitalizations, and 69 deaths. Similarly, in 2011, more than 5,500 foodborne outbreaks, causing more than 7,000 hospitalizations and 93 deaths, were reported in the European Union (EFSA-ECDC, 2013).

In US, from 1999 to 2008, ca. 47% of foodborne outbreaks were associated with meats (beef, pork, poultry, etc); among these more than 75% were attributed to *Clostridium perfringens* and *Salmonella* sp. strains (Batz, 2013).

The European Rapid Alert System for Food and Feed (RASFF) portal reported that, from January 2010 to June 2015, among over 16,000 notifications, 1035 regarded meat and meat products (other than poultry), with 87 referring to the presence of *Listeria monocytogenes* and, among these, 68 were associated with ready-to-eat meat products. Recently, a foodborne outbreak, caused by “rullepølse” (a type of rolled deli sausage served as cold cut meat), resulted in 12 deaths within 30 days of tests positive for *Listeria* (SSI, 2014; http://www.ssi.dk).

*Listeria monocytogenes* has been frequently isolated from food-processing environments (Chambel et al., 2007; Di Bonaventura et al., 2008; Fox et al., 2009) and this bacterium is a cause for concern in the chilled food industry due to its psychrotrophic nature (Norwood and Gilmour, 2001). More than 95% of human listeriosis are mainly due to serotypes 1/2a, 1/2b, and 4b (Swaminathan and Gerner-Smidt, 2007; Scallan et al., 2011). Differences in serotypes, the occurrence of several virulence factors and the long incubation period of invasive infection cause a high case-fatality rate (20–30%) in specific groups of consumers such as pregnant women, neonates and immunocompromised patients (Rocourt et al., 2003). However, also when the concentration of viable *L. monocytogenes* cells in foods is low, phenomena as post-process contamination and growth under refrigerated conditions are usually considered sufficient to cause human listeriosis (Wang and Orsi, 2013).

*Listeria monocytogenes* can rapidly form biofilms on food-contact surfaces such as plastic, polypropylene, rubber, stainless steel, and glass (Hood and Zottola, 1997; Silva et al., 2008). The inherent nature of a biofilm hinders the absorption of chemicals, which makes *L. monocytogenes* less susceptible to cleaning operations (Stopforth et al., 2002) improving the persistence of strains within food-processing premises (Norwood and Gilmour, 2001; Weiler et al., 2013). For these reasons, the Commission Regulation 2073/(Commission Regulation (EC), 2005) on microbiological criteria for foodstuffs requires that *L. monocytogenes* has to be absent in 25 g of food.

As recently reviewed by Ngadi et al. (2012), physical methods, chemical preservatives, and biopreservation are able to assure microbiological safety of foods even though they cause undesirable changes in the flavor, texture, and nutrient composition of foods. Emerging techniques for the control of foodborne pathogens are being studied to preserve the food freshness in terms of health-promoting bioactive compounds and organoleptic characteristics. In particular, a 600-W microwave treatment was able to achieve a 7-log reduction of *L. monocytogenes* in artificially ready to eat beef frankfurters within 12–15 min (Huang, 2005). Conversely, controlled radio frequency energy poorly affected *L. monocytogenes* inoculated in meat balls (Schlisselberg et al., 2013). Similarly, the application of pulsed electric fields resulted in low foodborne inactivation in foods, although it produced an increase in microbial sensitivity to following heat treatments (Hermawan et al., 2004; Lado et al., 2004). The application of high hydrostatic pressure processing resulted in weak inactivation of *L. monocytogenes* in cold-smoked salmon (Lakshmanan and Dalgaard, 2004), whereas when it was combined with nisin the listericidal effect increased in RTE cured meat products (Hereu et al., 2012).

Ozone (O_3_) is a powerful oxidant with high reactivity, penetrability, and spontaneous decomposition to non-toxic compounds (Miller et al., 2013). It has powerful antimicrobial properties (Gurley, 1985) and reacts with cells by attacking cell membranes, resulting in lysis of carbon–carbon double bonds in the membrane, causing cell lysis, and death. Treatment of meat samples contaminated with foodborne pathogens with either gaseous or aqueous ozone usually results in very low decontamination levels. The comparison of three different ozone treatments (gaseous, aqueous, and humidified) at three concentrations and times of exposure showed that the gaseous treatment was the most effective at reducing *L. monocytogenes* spread onto cured ham slices (Julson et al., 2001); in this case, the reduction of viable cell count was again probably limited due to the presence of interfering organic matter. Piachin and Trachoo (2011) reported that the introduction up to 1000 mg h^-1^ of ozone into an airtight plastic bag, containing artificially contaminated fresh pork meat, was unable to reduce listeriae load during 15 days of storage at 8°C.

The use of high ozone concentration (154 × 10^-6^ kg m^-3^) for three hours reduced only 1 log10 cycles of non-pathogenic *Escherichia coli* inoculated in beef sample (Coll Cárdenas et al., 2011). A similar low killing rate was obtained after exposing natural hog casing, contaminated with a different non-pathogenic *E. coli* strain, to ozoneated water (ca 7 mg L^-1^; Benli et al., 2008).

More recently, 2 × 10^6^ CFU/g of *L. monocytogenes* on chicken samples resulted inactivated after 9 min of gaseous ozone treatment at 33 mg min^-1^ in a small ozonation chamber (ca 3.5 L); however, no results were reported with regard to meat quality after being subjected to such severe ozonation (Muthukumar and Muthuchamy, 2013).

Results from study of Güzel-Seydim et al. (2004) suggested that high levels of protein or fat protected both bacterial spores and vegetative cells when exposed to ozonated water for a short time (10 min).

Cross-contamination of foodborne pathogens from inert surfaces to foods has been reported diffusely (Kusumaningrum et al., 2003; Lin et al., 2006; Wilks et al., 2006). Lin et al. (2006) demonstrated that *L. monocytogenes* could be transferred from a contaminated slicer onto meats and the pathogen survived better on uncured oven-roasted turkey than on salami or bologna with preservatives.

On the other hand, Nicholas et al. (2013) reported a significant reduction in *L. monocytogenes* viable cell load after ozone treatment of contaminated contact surfaces. The viable cell load of one single *L. monocytogenes* strain, deposited onto stainless steel, granite and polypropylene coupons, was found significantly reduced up to 3.42 mean log CFU cm^-2^ at ozone concentration of 96.3 mg m^-3^ (45 ppm). However, this concentration value is usually considered detrimental to human health and can cause unacceptable worsening of meat nutritional quality (Coll Cárdenas et al., 2011).

Many governmental and private institutions have considered the effect of long-term human exposure to ozone. Usually, the threshold limit of ozone exposure, calculated as 8 h day^-1^ (40 h week^-1^) average exposure (TLV-TWA value), is considered to be 0.1 ppm (0.2 mg m^-3^). Increasing exposure and concentration of ozone can result in headache, eye, nose, throat and respiratory irritation, lung damage with chronic respiratory disease, edema, and hemorrhage (OSHA, 2012).

The aim of this work was to evaluate the antimicrobial efficacy of gaseous ozone at low concentrations (compatible with human exposure) against foodborne pathogens, with particular reference to *L. monocytogenes*, contaminating cold storage food-contact surfaces.

## Materials and Methods

### Bacterial Strains, Culture Conditions, and Inoculum on Food-Contact Surfaces

The foodborne pathogenic strains used in this study, growth media, and culture conditions are reported in **Table 1**. All substrates were purchased from Biolife Italiana srl, Milan, Italy. All materials used in this study (stainless steel, expanded polystyrene, glass, and polypropylene) were provided by a meat-processing enterprise (Dodaro S.p.A, Spezzano Albanese, Italy) and were regulated under the Framework Regulation (EC) 1935/(Commission Regulation (EC), 2004) on materials and articles intended to come into contact with food. Stainless steel, polypropylene, and glass coupons (about 5 cm^2^) were sterilized by autoclaving at 121°C for 15 min before use whereas expanded polystyrene coupons (about 5 cm^2^) were dipped in hypochlorite solution (400 mg L^-1^) for 10 min, vigorously washed in 100 ml of MQ autoclaved water for 5 min, and then dried in a sterile biohazard cabinet.

**Table 1:** Indicator strains, culture, and selective media and related growth conditions.

Indicator strain^a^	Culture media^b^ and growth conditions	Selective media^c^ and growth conditions
**Gram-negative**		
*Pseudomonas aeruginosa* DSM 939	PCB, 30°C, 150 rpm, 16 h	PSA + CFC supplement, 37°C, 24 h
*Escherichia coli* ATCC 35401	PCB, 37°C, 150 rpm, 16 h	TBX, 37°C, 24 h
		
**Gram-positive**		
*Staphylococcus aureus* NCTC 8325	BHI, 37°C, 150 rpm, 16 h	BP + egg yolk tellurite emulsion; 37°C, 24 h
*Listeria grayi* DSM 20601^t^	BHI, 37°C, 150 rpm, 16 h	ALOA + Selective and Enrichment Supplement, 37°C, 24 h
*L. innocua* DSM 20649^t^		
*L. ivanovii* ssp. *ivanovii* DSM 20750^t^		
*L. welshimeri* DSM 20650^t^		
*L. monocytogenes* LMG 23774, LMG 23775, LMG 23905, LMG 23192, DSM 20600^t^. LMG 10470, LMG 23189		

*^a^DSM, Deutsche Sammlung von Mikroorganismen und Zellkulturen, Braunschweig, Germany; ATCC, American type culture collection, LGC Standards, Sesto San Giovanni, Italy; NCTC, National Collection of Type Cultures, Salisbury United Kingdom; LMG, Belgian Coordinated Collection of Microorganisms, Laboratorium voor Microbiologie Universiteit Gent, Gent, Belgium.*

*^b^PCB, plate count broth; BHI, brain heart infusion.*

*^c^PSA, Pseudomonas selective agar; TBX, tryptone bile x-gluc agar; BP, Baird Parker; ALOA, agar Listeria Ottaviani and Agostit:type strain.*

All coupons were tainted following the AOAC 961.02 Method (AOAC International, 2009). Working microbial cell suspensions were prepared from frozen cultures (–80°C) as reported in **Table 1**. Fresh grown microbial cells, at an OD_600_ value of 0.3 ± 0.10 (ca. 8 log CFU ml^-1^) were harvested by centrifugation (5000 × *g* for 5 min), washed twice and resuspended in sterile saline solution. Twenty microliter of this suspension was distributed drop by drop onto coupons and spread uniformly across the area with a sterile loop, excluding 2 mm of the edge. Coupons were allowed to dry in a sterile biohazard cabinet for ca. 30 min and then placed in a sterile ventilated Petri dish. These dishes, classified as biological substance category B, were marked with the code UN 3373 (WHO/HSE/GCR/2012.12, 2012), and transported as described in the WHO/EMC/97.3 (1997) for local surface transport. During incubation, only admitted personnel had access to the cold storage chamber.

### Ozone Generation

Food contact surfaces were incubated, depending on the experiment, for 1 week in a pilot cold storage chamber (M. G. di Narducci Lucia, Capurso, BA, Italy) having an internal volume of 3.36 m^3^ endowed with continuous air ventilation and set at 4°C. Ozone was generated using an OGS358 apparatus (OEC s.n.c., Pedrengo, BG, Italy) endowed with an internal semiconductor SnO_2_ probe for controlling ozone concentration in the volume of the cold chamber (concentrations by volume: 1 ppm O_3_ = 2.14 mg m^-3^).

Coupons were incubated 2 m from a gas inlet; ozone concentration was monitored, near the coupons, using a specific data logger (Oneset Hobo datalogger, Cape Cod, MA, USA), every 10 min. The surface-attached bacteria were subjected to ozone concentrations of 0, 1.07, and 3.21 mg m^-3^.

Air temperature and relative humidity were measured, near the coupons, every 10 min; data were stored on an iButton^®^ temperature/humidity logger model DS1923 (Maxim Integrated, San Jose, CA, USA).

### Enumeration of Survivors

Immediately before cold incubation (ca. 45 min after inoculation of coupons), and on the sampling days specified for each experiment (in the presence or absence of ozone), the coupons were evaluated for viable microbial cells. Recovery of viable cells from each surface was obtained by vortexing (for 1 min at maximum speed) coupons dipped in 40 ml of sterile saline solution. This cell suspension was decimally diluted and plated (0.1 ml) onto selective media (**Table 1**). The detection limit of this plating technique was calculated in 400 CFU coupon^-1^. In order to improve this detection limit, an enrichment culture procedure was carried out: 10 ml of saline solution, used to detach bacteria from the coupons, was inoculated in 90 ml of specific culture medium, and flasks were incubated at 37°C for 48 h; then, a loopful of suspension was plated on selective chromogenic medium and incubated as reported in **Table 1**. The detection limit of this procedure was estimated as 4 CFU coupon^-1^. When no specific colonies were detected after enrichment and plating on selective chromogenic media, populations were indicated as not detected (ND).

### Experimental Design and Evaluation of the Antimicrobial Effect of Gaseous Ozone Treatment

**FIGURE 1:**
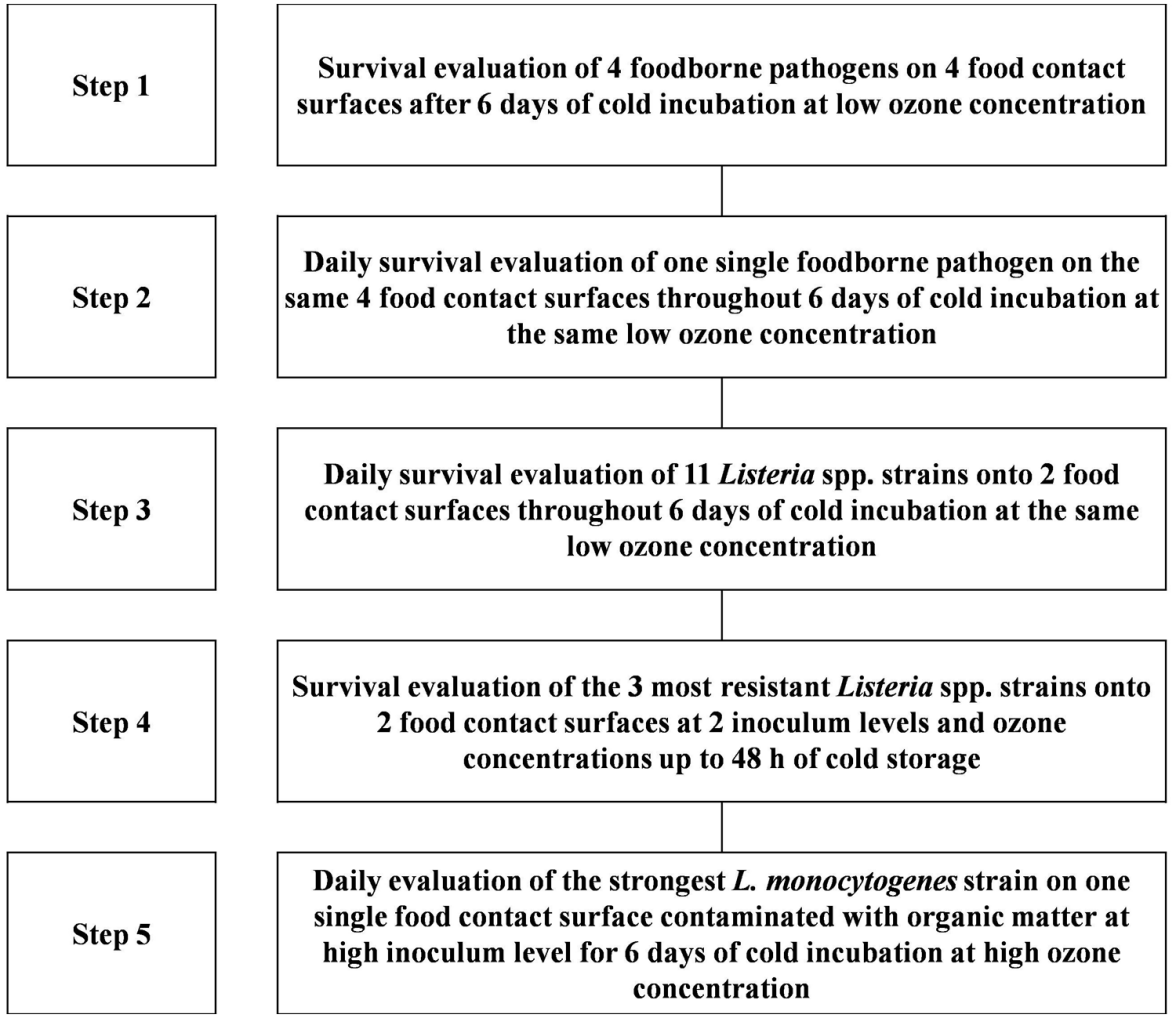
**Graphical scheme of the experimental plan carried out in the present work**.

The experimental flowchart carried out in the present work is reported in **Figure 1**.

#### Survival of Foodborne Pathogens on Food Contact Surfaces

At first, four indicator strains (*L. monocytogenes* LMG 10470, *E. coli* ATCC 35401, *Pseudomonas aeruginosa* DSM 939, and *Staphylococcus aureus* NCTC 8325), laid on four food-contact surfaces (glass, polypropylene, stainless steel, and expanded polystyrene), were evaluated for their survival after 6 days at 4°C and in the presence of 1.07 mg m^-3^ of ozone (**Figure 1**, Step 1).

On the basis of the results, the subsequent experiment was carried out with *L. monocytogenes* LMG 10470 evaluating daily its resistance to the same ozone treatment throughout 6 days of incubation at 4°C. The above mentioned inoculated surfaces, incubated under the same conditions without ozone, were used as negative controls (**Figure 1**, Step 2).

#### Selection of the Most Resistant Strain/Surface Combination to Ozone Treatment

Afterward, four type strains of *Listeria* sp. and seven strains of *L. monocytogenes* (listed in **Table 1**), laid on stainless steel and expanded polystyrene, were subjected to 1.07 mg m^-3^ ozone treatment at 4°C for 6 days and cell survival was evaluated daily. (**Figure 1**, Step 3).

On the basis of the previous results, in order to evaluate the effect of different concentrations of ozone on viable cell populations, *L. monocytogenes* LMG23775 and 23192 and *L. innocua* DSM20649 were laid onto stainless steel and expanded polystyrene coupons and incubated, up to 48 h at 4°C, at two ozone concentrations, 1.07 and 3.21 mg m^-3^, respectively. Two different levels of inoculum, herein referred to as low (L) and high (H), were obtained (diluting or concentrating 10 times a fresh culture of ca. 8 log CFU ml^-1^) and used. Cell survival was evaluated daily for 48 h with the enrichment procedure described in the Section “Enumeration of Survivors” (**Figure 1**, Step 4).

#### Effect of Ozone Treatment on *L. monocytogenes* LMG 23775 Laid on Dirty Expanded Polystyrene Coupon

In order to evaluate the effect of gaseous ozone treatment on *L. monocytogenes* LMG 23775, laid on dirty expanded polystyrene coupon, the pathogen was tested following the UNI EN 13704 (2005) standard, replacing the interfering substance (bovine albumin solution 3 g L^-1^) with a raw meat extract, obtained by blending (at the highest speed and for 2 min) 25 g of pork meat in 225 ml of sterile saline solution. The watery meat mixture was then filtered through cotton gauze. One milliliter of LMG2377 growth culture was washed in sterile saline solution and resuspended in 100 ml (high inoculum level) of this meat extract. Twenty microliter of the inoculated meat extract was laid on expanded polystyrene coupon, resulted the most resistant surface to ozone, and the pathogen was ozonated for 1 week at 3.21 mg m^-3^. Negative controls were coupons tainted with 20 μl of the same meat extract without LMG2377 cells, and incubated under the same conditions in the presence and in absence of ozone (**Figure 1**, Step 5).

Besides, both meat extract and cell suspensions from the coupons were evaluated every day for 6 days for *Listeria* population, total mesophilic aerobic bacteria as aerobic plate count (APC, on Plate Count agar; Ryser and Schuman, 2013), *E. coli* and *Enterobacteriaceae* (on TBX agar) and coagulase-positive and -negative staphylococci (CNS or, on Baird Parker agar supplemented with egg-yolk tellurite emulsion). All the assays were repeated three times independently.

### Statistical Analyses

Ozone treatments were independently performed in triplicate (*n* = 3). The concentration of viable cells in samples was calculated as the average number of colonies found for each decimal dilution, corrected by the dilution factor and expressed as log CFU mL^-1^ or log CFU coupon^-1^ ± SD. Average microbial populations were analyzed by one-way analysis of variance (ANOVA); the significance of differences (*P* ≤ 0.05) between mean values was evaluated by the Tukey test. In addition, by applying a two-way ANOVA (*P* ≤ 0.05), independent effects and interactions of the main factors (time of storage and treatment) on the microbial populations were evaluated. Multiple comparisons among individual means within the same microbial group were made by Fisher’s least significant difference (LSD) *post hoc* test after rejecting the homogeneity of their variances using the Levene test with an α level of *P* ≤ 0.05.

Statistical analyses were performed with the Microsoft Excel software, implemented with the statistical analysis add-in (Microsoft Corporation, Redmond, WA, USA).

## Results

### Evaluation of Incubation Conditions

**FIGURE 2:**
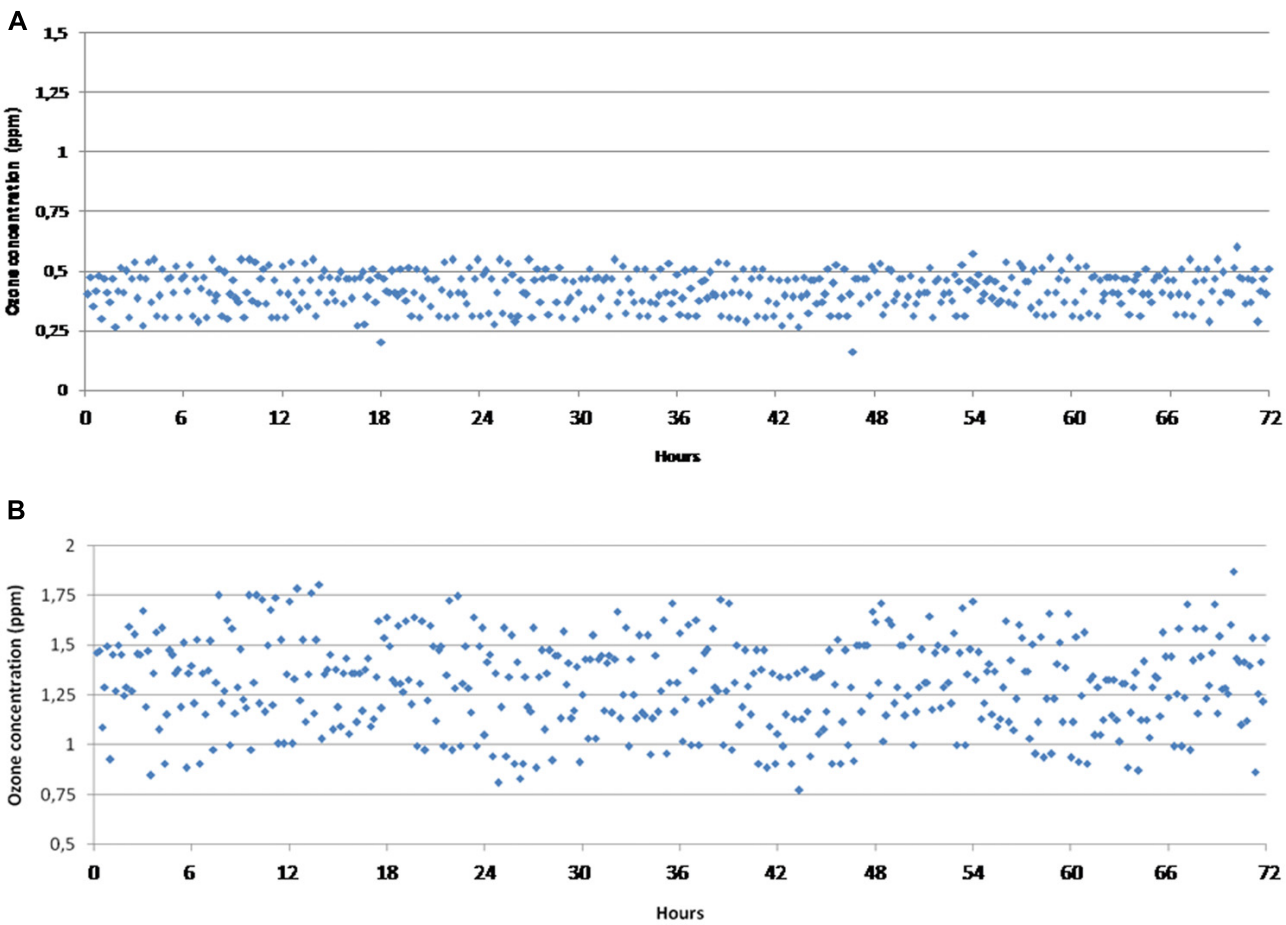
**Ozone concentration recorded in the cold storage chamber for 3 days of continuous flow with the control system set at 1.07 mg m^-3^ (A) and 3.21 mg m^-3^ (B)**.

In **Figure 2** the effect of ozone concentration in the cold storage chamber throughout 3 days of continuous flow with the control system set at 1.07 mg m^-3^ (panel A) and 3.21 mg m^-3^ (panel B), respectively, is shown. As recorded by different data loggers, temperature was stable at 3.3 ± 0.3°C whereas the average RH value was 64.2 ± 4.8 %. The ozone concentration resulted affected by weak fluctuations at both ozone concentrations throughout 3 days of continuous flow. When the ozone generator was set up at 1.07 mg m^-3^, the ozone concentration near the coupons ranged from 0.342 to 1.28 mg m^-3^ and was, on average, 0.90 ± 0.17 mg m^-3^. At 3.21 mg m^-3^, the average concentration was 2.80 ± 0.49 mg m^-3^ whereas values changed from 1.65 to 4.00. Oscillations are in line with the generation of ozone flowing in the refrigerator volume with the concentration control system 90 cm from the ozone inlet.

### Antimicrobial Effect of Gaseous Ozone Treatment

#### Survival of Foodborne Pathogens on Food Contact Surfaces

At first, the adaptation of the foodborne pathogens *E. coli* ATCC 35401, *P. aeruginosa* DSM 939, *S. aureus* NCTC 8325 and *L. monocytogenes* LMG 10470 to cold environment, when laid on four different food-contact surfaces, and also in the presence of 1.07 mg m^-3^ of ozone was evaluated (**Figure 1**, Step 1). As shown in **Table 2**, no viable cells, belonging to all tested strains, were recovered from all food contact surfaces after 6 days of gaseous ozone treatment.

**Table 2:** Effect of continuous gaseous ozone flow at 1.07 mg m^-3^ on the survival of foodborne pathogen cells (in mean log CFU coupon^-1^) laid on four surfaces, after 6 days at 4°C.

Strain	Stainless steel			Polypropylene			Glass			Expanded polystyrene		
	
	T0	T6		T0	T6		T0	T6		T0	T6	
		O_3_	C		O_3_	C		O_3_	C		O_3_	C
*L. monocytogenes* LMG 10470	6.42 ± 0.14^a^	ND	6.14 ± 0.51^a^	6.22 ± 0.26^a^	ND	6.08 ± 0.11^a^	6.70 ± 0.27^a^	ND	5.46 ± 0.16^b^	6.34 ± 0.25^a^	ND	5.21 ± 0.28^b^
*E. coli* ATCC 35401	5.36 ± 0.36^a^	ND	ND	5.91 ± 0.33^a^	ND	3.60 ± 0.24^b^	5.56 ± 0.36^a^	ND	ND	5.62 ± 0.90^a^	ND	ND
*P. aeruginosa* DSM 939	7.11 ± 0.22^a^	ND	ND	7.18 ± 0.90^a^	ND	3.30 ± 0.16^b^	7.44 ± 0.48^a^	ND	ND	7.81 ± 0.11^a^	ND	ND
*S. aureus* NCTC 8325	5.56 ± 0.05^a^	ND	ND	5.70 ± 0.63^a^	ND	ND	5.27 ± 0.32^a^	ND	ND	5.61 ± 0.44^a^	ND	ND

*C-inoculated but un-ozonated coupons (control).*

*ND-not detected after the enrichment procedure described in the Section “Enumeration of Survivors.”*

*Values are means (±SD). Within rows, means followed by different lowercase letters are significantly different (P < 0.05).*

In the absence of ozone (negative controls), *E. coli* ATCC 35401, *P. aeruginosa* DSM 939, and *S. aureus* NCTC8325 were unable to survive at 4°C after 6 days, when deposited on stainless steel, glass, and expanded polystyrene coupons whereas a partial survival was displayed by ATCC 35401 and DSM 939 on polypropylene coupons only after the enrichment procedure (described in Section “Enumeration of Survivors”), as shown in **Table 2**.

Unlike the other strains, *L. monocytogenes* LMG 10470 resisted under cold storage conditions (with an average reduction of 0.7 log CFU coupon^-1^) on all the four food-contact surfaces, even though differences in survival were found.

However, as shown in **Table 2**, no viable cells, belonging to all tested strains, were recovered from all food contact surfaces after 6 days of gaseous ozone treatment.

On the basis of these results, in the subsequent experiment the impact of gaseous ozone flow (1.07 mg m^-3^) was evaluated daily for 6 days of cold storage only on LMG 10470 (**Figure 1**, Step 2). After 24 h, no viable LMG 10470 cells were harvested from glass and polypropylene coupons, whereas viable cells (at an average of 4.08 log CFU coupon^-1^) were found on stainless steel and expanded polystyrene coupons, suggesting that these surfaces provided some protection to microbial cells (data not shown). However, on the same coupons, no viable colonies were retrieved after an additional 24 h of incubation also after the enrichment procedure. All coupons failed to show viable cells from the third to the sixth day of incubation under continuous 1.07 mg m^-3^ ozone treatment (data not shown).

#### Selection of the Most Resistant Strain/Surface Combination to Ozone Treatment

The subsequent experiment was carried out to verify the existence of a strain-specific resistance during cold storage and ozone treatment (4°C, 1.07 mg m^-3^) among the 11 *Listeria* sp. strains listed in **Table 1** (**Figure 1**, Step 3). Since ozone treatment resulted ineffective to eradicate LMG 10470 on stainless steel and expanded polystyrene coupons within 24 h of treatment (see previous results), these surfaces were chosen for further experiments.

As shown in **Table 3**, all strains survived during 6 days of incubation on both untreated-ozone surfaces, recording an average reduction of 2 log cycles. *Listeria* strains, initially laid at an average value of 6.43 ± 0.29 log CFU coupon^-1^, showed a different survival rate depending on the kind of surface treated with gaseous ozone (**Table 3**). After 1 day of treatment, only *L. grayi* DSM 20601 and *L. monocytogenes* LMG 23192 displayed viable cells (on average, 5.12 log CFU coupon^-1^) on stainless steel, whereas *L. monocytogenes* LMG 23775 was found only after the enrichment procedure described in Section “Enumeration of Survivors.” An extension of the incubation period up to 48 h completely eradicated these three strains from stainless steel coupons. On expanded polystyrene, viable cell counts belonging to all *Listeria* except for *L. monocytogenes* DSM 20600^t^ were found only after 24 h of ozone treatment; the extension of incubation for an additional 24 h eradicated all *Listeria* viable cells with the exception of LMG 23192 and DSM 20649^t^. As found for the stainless steel surface, the extension of the incubation period killed all listeriae on expanded polystyrene coupons (**Table 3**). Since the concentration of viable cells was calculated only when strains were laid on expanded polystyrene coupons, the strain-specific influence on survival under gaseous ozone treatment was obtained from these data (**Table 3**). The killing rate of 1 day treatment with ozone ranged, comparing ozone-treated microbial populations with those exclusively cold-stored (untreated control), from 7% (DSM 20649) to 100% (DSM 20600^t^).

**Table 3:** Effect of continuous gaseous ozone flow at 1.07 mg m^-3^ on the survival of foodborne pathogen cells (in mean log CFU coupon^-1^) laid on stainless steel and expanded polystyrene for 6 days (T1–T6) at 4°C.

Strain	T1		T2		T3–6	
	
	Stainless steel	Expanded polystyrene	Stainless steel	Expanded polystyrene	Stainless steel	Expanded polystyrene
*L. grayi* DSM 20601^t^	5.15 ± 0.05	3.25 ± 0.07^a^	ND	ND	ND	ND
*L. innocua* DSM 20649^t^	ND	6.06 ± 0.05^b^	ND	+	ND	ND
s *L. ivanovii* ssp. *ivanovii* DSM 20750^t^	ND	4.41 ± 0.21^c^	ND	ND	ND	ND
*L. welshimeri* DSM 20650^t^	ND	5.16 ± 0.22^d^	ND	ND	ND	ND
*L. monocytogenes* DSM 20600^t^	ND	ND	ND	ND	ND	ND
*L. monocytogenes* LMG 10470	ND	5.40 ± 0.44^d^	ND	ND	ND	ND
*L. monocytogenes* LMG 23189	ND	5.06 ± 0.14^d^	ND	ND	ND	ND
*L. monocytogenes* LMG 23192	5.12 ± 0.14	4.36 ± 0.32^c^	ND	2.60 ± 0.12	ND	ND
*L. monocytogenes* LMG 23774	ND	5.21 ± 0.20^d^	ND	ND	ND	ND
*L. monocytogenes* LMG 23775	+	5.06 ± 0.32^d^	ND	ND	ND	ND
*L. monocytogenes* LMG 23905	ND	4.86 ± 0.42^cd^	ND	ND	ND	ND

*ND-Viable cells of target microorganism not detected after the enrichment procedure.*

*+-Viable cells of target microorganism found on ALOA selective agar plates after the enrichment procedure.*

*Values are means (SD). Within columns, means followed by different lowercase letters are significantly different (P < 0.05).*

These assays indicated that ozone antimicrobial activity partially depended on the *Listeria* strains tested. LMG 23192 resulted the most resistant *L. monocytogenes* strain on both stainless steel and expanded polystyrene surfaces and, in addition, it was the only strain still showing countable viable cells after 48 h of cold ozone treatment on expanded polystyrene coupons. *L. innocua* DSM 20649^t^ resulted the most resistant strain on expanded polystyrene coupons within the first 24 h and partially viable at T2 (**Table 3**).

**FIGURE 3:**
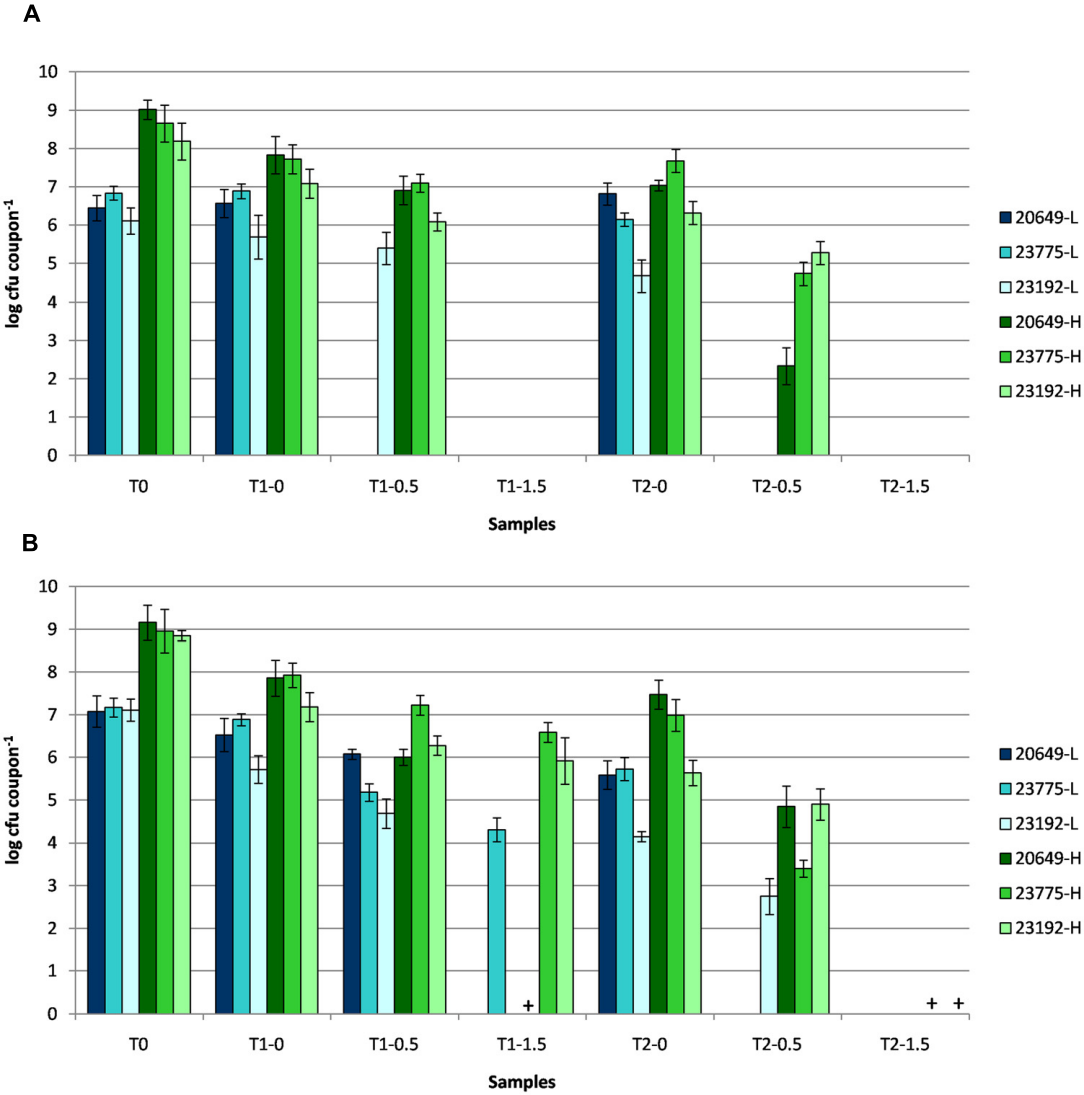
**Enumeration of viable cells (in log CFU coupon^-1^) of *L. innocua* DSM 20649, *L. monocytogenes* LMG 23775, and LMG 23192 laid at low and high inoculum levels (L and H) onto stainless steel (A) and expanded polystyrene (B) coupons, for 2 days (T1–T2) of cold incubation at 0 mg m^-3^ (Control), 1.07 mg m^-3^ (0.5) and 3.21 mg m^-3^ (1.5) ozone concentration.** Samples showing viable cells after the enrichment procedure are indicated with “+.”

As resistance could partly depend on the initial microbial load of pathogens subjected to ozone treatment, in the subsequent experiment *L. monocytogenes* LMG 23775, LMG 23192, and *L. innocua* DSM 20649^t^, showing the best survival in the previous experiment, were assayed at two different levels of inoculum, i.e., L (ca. 7 log CFU coupon^-1^) and H (ca. 9 log CFU coupon^-1^), at 1.07 and 3.21 mg m^-3^ ozone concentrations on both stainless steel and expanded polystyrene surfaces (**Figures 3A,B**). As shown in **Figure 3**, when coupons with L inoculum were ozonated at 1.07 mg m^-3^ (0.5) ppm good levels of resistance were observed for all strains on expanded polystyrene (**Figure 3B**), and in particular, for the strain *L. monocytogenes* LMG 23192 that amounted to 2.75 ± 0.42 log CFU coupon^-1^ after 48 h of treatment, confirming the results obtained previously. This strain was also the only strain surviving on a stainless steel surface, but only for the first part (24 h) of the treatment (**Figure 3A**).

The increase in the inoculum level resulted in a better survival: under these conditions (H inoculum and 1.07 mg m^-3^), all strains deposited onto stainless steel coupons (**Figure 3A**) resisted the ozone treatment up to 48 h, even though 4.74, 2.92, and 1.04 log reductions were recorded for DSM 20649^t^, LMG 23775 and LMG 23192 strains, respectively (**Figure 3**).

The survival of listeriae in the absence of ozone treatment on expanded polystyrene coupons at H inoculum level (negative control samples) showed an average log reduction of 1.33 ± 0.03 and 2.29 ± 0.13 after 24 or 48 h of cold storage, respectively (**Figure 3B**). Compared to these values, the influence of ozone treatment (1.07 mg m^-3^) resulted in an increase in average log reduction to 2.48 ± 0.13 and 5.89 ± 0.29 for the same sampling days.

Ozone concentration of 3.21 mg m^-3^ eradicated all tested listeriae laid on stainless steel coupons after just 1 day of treatment. Differently, on expanded polystyrene, strains LMG 23192, and LMG 23775, treated with 3.21 mg m^-3^ of ozone, resisted better than DSM 20649^t^, whose viability was found only after the enrichment procedure. The extension in the incubation period improved the antimicrobial efficacy of the ozone treatment for these coupons, showing viable cells for *L. innocua* DSM 20649^t^ and *L. monocytogenes* LMG 23775 only after the enrichment procedure. The two-way ANOVA statistical analysis demonstrated that the increase in ozone concentration, the extension in the treatment period as well as their interaction on antimicrobial efficacy were statistically significant (*P* ≤ 0.05).

#### Effect of Ozone Treatment on *L. monocytogenes* LMG 23775 Laid on Dirty Expanded Polystyrene Coupon

Cross-contamination of foodborne pathogens from inert surfaces to foods, and vice-versa, are well known. In the present work, validation of ozone treatment (4°C, 1 week, 3.21 mg m^-3^ ozone concentration) was carried out against LMG 23775, the most resistant *L. monocytogenes* strain tested, at the H inoculum level (8.79 ± 0.16 log CFU coupon^-1^), in the presence of interfering meat-extract organic matter laid on expanded polystyrene coupon, the food-contact surface which was most difficult to disinfect (**Figure 1**, Step 5).

**FIGURE 4:**
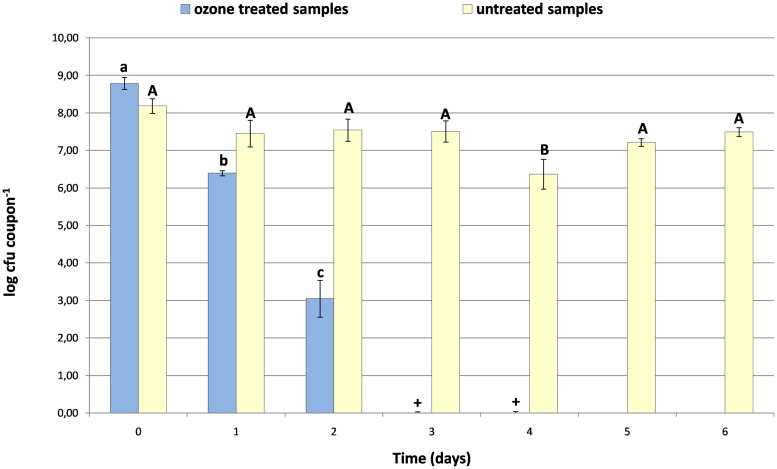
**Effect of continuous gaseous ozone flow (3.21 mg m^-3^ = 1.5 ppm) on the survival of *L. monocytogenes* LMG 23775, resuspended in meat extract and laid on expanded polystyrene coupons for 6 days of incubation at 4°C.** Bars represent means (with their SD) with different capital or lowercase letters expressing significant differences from one-way ANOVA analysis (*P* < 0.05) for untreated or ozone-treated samples, respectively. Samples showing viable cells after the enrichment procedure are indicated with “+.”

In the absence of ozone, a cell concentration of LMG 23775 suspended in meat extract was found rather stable until the sixth day of cold incubation (average reduction of 1.3 log CFU coupon^-1^), whereas ozonation significantly reduced the number of viable LMG 23775 cells after just 24 h of cold incubation (**Figure 4**).

LMG 23775 load was 3.05 ± 0.49 log CFU coupon^-1^ on two out of three contaminated coupons; the third coupon showed viable cells only after the enrichment procedure. The same behavior was observed for all coupons at the third and fourth day of treatment. No viable *L. monocytogenes* LMG 23775 cells were found from the fifth day of treatment onwards (**Figure 4**).

Organic matter on food-contact surfaces are contaminated by several types of microorganisms that could affect the efficacy of ozone in removing *L. monocytogenes*. For this reason, we evaluated the fate of contaminating microflora when un-sterile meat extract dirtied expanded polystyrene. Meat extract amounted to 3.63 ± 0.73 log CFU g^-1^ of APC, CNS and *Enterobacteriaceae* mixed bacterial populations. APC at 2.9 log CFU coupon^-1^ was still found as the only microbial population from meat extract that survived on untreated (cold-stored) coupons, but only after 24 h of storage. None of these microbial populations was enumerated on either ozonated or untreated un-inoculated coupons from days 2 to 6 of the experiment. These results suggest that the influence of bacteria naturally occurring in meat extract was negligible on the efficacy of ozone treatment.

## Discussion

It has been widely demonstrated that ozone treatments are unable to completely eradicate foodborne pathogens from meats and meat products (Julson et al., 2001; Benli et al., 2008; Coll Cárdenas et al., 2011; Piachin and Trachoo, 2011). For this reason, we evaluated the efficacy of gaseous ozone treatment in reducing the survival of foodborne pathogens on food-contact surfaces in order to control cross-contamination phenomena. After some assays on some foodborne pathogens (**Tables 2** and **3**; **Figure 3**), the experimental activity focused only on *L. monocytogenes* that, thanks to its psychrotrophic nature, was considered the foodborne pathogen potentially most responsible for cross-contamination of food-contact surfaces under refrigerated conditions. Therefore, strains belonging to this species were considered more useful to define a set of parameters for decontaminating materials used in food-processing plants (**Figure 4**). Experimental activities showed that listeriae had different behaviors when deposited on different surfaces. Adherence, viability and survival of microbial cells on an inert surface, and in the absence of nutritional supplementation, depend on the interaction between microbial strains and the physico-chemical characteristics of the material. Usually the hydrophobicity or else the hydrophilicity of surface as well as its roughness are considered the main factors. Previous studies showed that *L. monocytogenes* can adhere to different food-use materials (including metals, rubbers, and polymers; Mafu et al., 1990; Beresford et al., 2001). Later, Silva et al. (2008) demonstrated that *L. monocytogenes* adhered most tightly to stainless steel, glass, and then to polypropylene surfaces; however, viable cell loads from polypropylene and glass were higher (close to 100%) than those from stainless steel despite the low level of adhesion.

In the absence of experimental assays designed to study the adherence of strains, we found that ozonated microbial cells survived better when deposited onto stainless steel and expanded polystyrene than onto polypropylene and glass. These results differ from those of Mafu et al. (1990) and Nicholas et al. (2013), who found a higher survival rate of *L. monocytogenes* on polypropylene rather than on stainless steel after their treatment with sanitizing agents or 96.3 mg m^-3^ (45 ppm) gaseous ozone, respectively. Differences in target strain as well as in parameters of experiments could be responsible for the differences found.

To the best of our knowledge, no studies have described so far the adhesion and viability of *L. monocytogenes* cells when laid on expanded polystyrene, a common material used in food packaging and storage.

Our work is the first report describing the survival of *L. monocytogenes* under cold storage and ozone treatment on this surface. Even though the hydrophobicity of expanded polystyrene prevents the absorption of water, empty spaces between polyhedra can accumulate water under vapor or liquid phase (Horvath, 1994). In order to react with microbial membranes, ozone needs to be dissolved in water, a process involving different steps that potentially decompose ozone (Norton and Misiewicz, 2012), thus we can assume that higher rates of listeriae survival on expanded polystyrene could be due to the longer time needed for ozone to reach the antimicrobial concentration from gaseous to watery solution. However, we were unable to ascertain whether the increased resistance of *L. monocytogenes* on this polymer is also accompanied by biofilm formation that is usually involved in both adhesion and ozone resistance (Norwood and Gilmour, 2001; Stopforth et al., 2002; Di Bonaventura et al., 2008; Jordan et al., 2008; Nicholas et al., 2013).

It is well known that the ability of *L. monocytogenes* to adhere to food-contact surfaces depends on the material and strain and is affected by a high interstrain variability (Mafu et al., 1990; Scott and Bloomfield, 1990; Lundén et al., 2000; Silva et al., 2008), however, few studies evaluated the strain-specific response to gaseous ozone treatments of microbial cell loads on inert surfaces. Early experiments showed that Gram-negative bacteria were more sensitive than Gram-positive ones when laid onto stainless steel squares and exposed at 4.28 mg m^-3^ (2 ppm) ozone concentration for 4 h, although these conclusions derived only from experiments on four strains of different species (Moore et al., 2000). Similar results were obtained by Li and Wang (2003) using ozone concentrations from 1.28 to 34.24 mg m^-3^ (0.6 to 16 ppm) for a contact time from 30 to 150 min against vegetative cells and spores of one single strain of *E. coli* and *Bacillus subtilis*, respectively. In the present work, we found that *Listeria* sp. strains expressed different survival rates; however, under the experimental conditions tested, only *Listeria* strains survived on expanded polystyrene coupons (**Tables 2** and **3**). These results shed light on the link among strain, surface and ozone treatment parameters, thus experimental results from one single strain should be carefully evaluated, given that differences in ozone administration could impact heavily on the final results.

As concerns the influence on the killing rate of ozone concentration and duration of treatment, we found that both as well as their interaction influenced it significantly (**Figure 3**), in accordance with previous results (Moore et al., 2000; Julson et al., 2001; Li and Wang, 2003; Coll Cárdenas et al., 2011; Nicholas et al., 2013). In addition, we demonstrated that any increase in the amount of microbial cells to be treated required more severe treatments (**Figure 3**).

Besides, we found that the occurrence of organic matter (meat extract in this case) reduced the efficacy of ozone treatment (**Figure 4**) confirming data reported by other authors (Moore et al., 2000; Julson et al., 2001; Güzel-Seydim et al., 2004; Segat et al., 2014). In addition, our experiment was carried out under more difficult conditions (the more resistant *L. monocytogenes* strain was laid at high inoculum level on expanded polystyrene surface) and also in presence of other microorganisms contaminating meat extracts that could have influenced the adherence capabilities of *L. monocytogenes* LMG 23775 (**Figure 4**), as demonstrated for other strains on stainless steel coupons (Norwood and Gilmour, 2001).

The ozone concentration found to be useful to eradicate *L. monocytogenes* from inert surfaces (3.21 mg m^-3^ = 1.5 ppm) under the worst conditions needs to be carefully evaluated as it is higher than the TLV–TWA value; however, it is lower than 10.7 mg m^-3^ (5 ppm), the IDLH value considered as dangerous to life or health as established by the US National Institute for Occupational Safety and Health (NIOSH, 2014).

## Conclusion

This work showed that gaseous ozone treatment may be used in a cold storage chamber to eradicate *L. monocytogenes* viable cells contaminating food-contact surfaces although killing efficacy showed increasing difficulty of disinfection roughly following the scale glass ≥ polypropylene > stainless steel > expanded polystyrene. Independently from the strain-specific sensitivity, 2 days of continuous ozone flow at 1.07 mg m^-3^ were sufficient to control 5–6 log CFU level of contamination (**Figure 4**); even though contamination levels of food-contact surfaces could be affected by several factors (strain, biofilm formation, surface material, cleaning procedure, and so on) their artificial contamination with *L. monocytogenes* never displayed load higher than 4–5 log CFU coupon^-1^ (Mafu et al., 1990; Beresford et al., 2001). However, in the event of higher contamination levels (8–9 log CFU coupon^-1^) and in the presence of meat-derived interfering organic matter, the complete eradication of viable *L. monocytogenes* was achieved by increasing the ozone concentration to 3.21 mg m^-3^ and extending the cold treatment for 5 days (**Figure 4**).

Based on these results, the normal level of *L. monocytogenes* contamination of food-contact surfaces, also when they present organic matter, can be controlled by the application of low concentrations of gaseous ozone employed in a cold environment.

## Conflict of Interest Statement

The authors declare that the research was conducted in the absence of any commercial or financial relationships that could be construed as a potential conflict of interest.
